# Tumor Location May Independently Predict Survival in Patients With M0 Squamous Cell Carcinoma of the Penis

**DOI:** 10.3389/fonc.2022.927088

**Published:** 2022-07-05

**Authors:** Kai Li, Xiang Le, Jianqing Wang, Caibin Fan, Jian Sun

**Affiliations:** Department of Urology, The Affiliated Suzhou Hospital of Nanjing Medical University, Suzhou Municipal Hospital, Gusu School, Nanjing Medical University, Suzhou, China

**Keywords:** squamous cell carcinoma of the penis, tumor location, overall survival, penile carcinoma specific survival, prognostic factor

## Abstract

**Background:**

To determine the association between tumor location and both clinicopathological characteristics and the survival of patients with M0 squamous cell carcinoma of the penis (SCCP).

**Methods:**

Data of 455 patients diagnosed with M0 SCCP between 1975 and 2018 were collected from the Surveillance, Epidemiology, and End Results (SEER) database of the United States National Cancer Institute. The effects of tumor location on overall survival (OS) and penile carcinoma-specific survival (PCSS) were analyzed using the Kaplan–Meier method. The Cox proportional hazards regression model was used to determine the impact of tumor location on PCSS.

**Results:**

SCCP was more likely to occur in the prepuce or glans (90%). Although no significant difference was observed between the OS of patients with M0 SCCP in the prepuce or glans and those with M0 SCCP in the body of the penis (p = 0.307), the former had better PCSS (p = 0.024). Moreover, M0 SCCP in the prepuce or glans was also significantly associated with better PCSS in patients with advanced age (age ≥ 60 years, p = 0.011), other ethnicities (p = 0.003), T2–T4 stage (p = 0.036), larger tumors (≥3 cm, p = 0.001), no regional lymph nodes removed (p = 0.044), and radical surgery (p = 0.027). Multivariate analysis confirmed that tumor location is an independent prognostic factor for patients with M0 SCCP [hazard ratio (HR) 1.881, p = 0.026].

**Conclusions:**

Tumor location is an independent prognostic factor for patients with M0 SCCP, and tumors in the prepuce or glans portend better PCSS.

## Introduction

Although penile cancer is relatively uncommon, it is a serious health concern in some regions. The incidence rates vary considerably, with 0.2 cases per 100,000 men in the United States and similarly lower rates in other developed countries (1). In developing countries, the incidence is higher at 4.4 cases per 100,000 men (2).

Squamous cell carcinoma (SCC) is the predominant pathological subtype of penile cancer (3). SCC of the penis (SCCP) frequently arises in the glans, followed by the prepuce, both glans and prepuce, coronal sulcus, and the shaft, in that order (4). Although the exact pathological basis of SCCP is unknown, human papillomavirus (HPV) infection (4) and lack of circumcision (5) are the major risk factors. Studies show that the incidence of penile cancer is negligible in Israeli-born Jews, most likely due to the prevalent practice of circumcision (5, 6). Other risk factors include a history of genital warts, penile tears, numerous sexual partners (>30 in the lifetime), smoking, and smegma (7, 8). Due to psychological factors, insidious and non-specific initial symptoms, and lack of awareness of the condition, approximately 15% to 60% of the patients delay presentation for at least 1 year (8–10). Furthermore, 66% of the patients initially present localized disease (8). Penile-preserving surgery is the current treatment standard for early penile cancer due to good functional and cosmetic results (11). For invasive tumors, however, partial or total penectomy is the only option (12, 13).

Several biomarkers for SCCP have been identified, although they are not routinely available in diagnosis and treatment. Panic et al. demonstrated that the characteristic shift in stromal-epithelial caveolin-1 (CAV1) was correlated with tumor progression in SCCP (14). Hu et al. found that overexpression of inhibitor of DNA binding 1 (ID1) was significantly relevant to lymph node metastasis in SCCP (15). Moreover, higher expression of miR-223-3p, miR-107, and miR-21-5p predicts a poor prognosis in penile cancer (16).

Currently, there are increasing studies on the prognostic factors for SCCP. Ornellas et al. reported that the presence and extent of metastasis to the inguinal region lymph nodes were important prognostic factors for survival in patients with invasive SCCP (17). Likewise, Cubilla et al. identified high histological grade as an adverse pathological prognostic factor for SCCP (18). In our previous study as well, we found that the presence of lymphovascular invasion and larger tumors (≥3 cm) portended a poor prognosis for SCCP patients (19, 20). However, the prognostic significance of tumor location is still unclear. The aim of this study was to determine the association between tumor location and both clinicopathological characteristics and the survival of patients with M0 SCCP by using the Surveillance, Epidemiology, and End Results (SEER) database.

## Methods

### Study Population

We used SEER*Stat (version 8.3.9) to collect all data from the SEER database (accession number 15498-Nov2020), a national cancer surveillance program supported by the Surveillance Research Program (SRP) in the National Cancer Institute’s (NCI’s) Division of Cancer Control and Population Sciences (DCCPS) (21). Initially, data of 1,937 patients with penile cancer diagnosed between 1975 and 2018 were extracted, and the cases were filtered according to the following inclusion criteria: 1) histopathologically confirmed tumor and surgical resection, 2) tumors defined as pure SCCP on the basis of the “International Classification of Diseases-Oncology, 3rd edition” (ICD-O-3) with codes 8051–8052 and 8070–8075 (22), and 3) no distant metastasis. Finally, 455 patients were enrolled in the study.

The variables for each patient included demographic characteristics (age and ethnicity) and clinicopathological characteristics (T stage, lymph nodes status, grade, tumor size, regional lymph nodes removed, surgery, and tumor location). The patients were followed up to the day of death or until December 31, 2018.

### Statistical Analysis

Overall survival (OS) and penile carcinoma-specific survival (PCSS) were the main endpoints of this study. OS was calculated from the date of SCCP diagnosis to the date of death from any cause or last follow-up (19). PCSS was calculated from the date of SCCP diagnosis to the date of death due to SCCP or last follow-up (19).

A two-sided chi-square test was used to compare baseline clinicopathological characteristics of patients with different tumor locations. The Kaplan–Meier method was used to screen for statistically significant indicators associated with OS and PCSS and to calculate survival probabilities. The differences in survival rates were compared using the log-rank test. Then, the multivariate Cox proportional hazards regression model included the statistically significant indicators identified by Kaplan–Meier analyses to confirm independent predictors of PCSS and was used to generate hazard ratio (HR) and 95% CIs. Subsequently, we formulated a forest plot with prognostic factors using Excel 2019. Moreover, the plots of stratified Kaplan–Meier analyses were used to demonstrate the correlation between different tumor locations and PCSS in each stratified variable. Two-sided p-values <0.05 were considered statistically significant. All statistical analyses were performed using SPSS 22.0 (IBM Corporation, Armonk, NY, USA).

## Results

A total of 455 patients with M0 SCCP were included, of which 409 (90%) had prepuce/glans tumors and 46 (10%) patients had tumors in the body of the penis. During the study period, 253 (55.6%) patients had died, and SCCP was the cause of death in 89 patients. The median follow−up period was 56 months (range, 0–179).

As shown in **Table 1**, SCCP occurred predominantly in elderly men (age ≥ 60 years, 78%). Furthermore, men of each ethnicity were prone to SCCP in the prepuce or glans. The result also indicated that patients with M0 SCCP in the prepuce or glans had the highest percentage of Caucasian patients (82.4%, p = 0.014). In addition, patients with M0 SCCP in the body of the penis were more likely to develop lymph node metastases than those with prepuce/glans tumors (p = 0.001). However, there were no significant differences in age (p = 0.278), T stage (p = 0.853), grade (p = 0.134), tumor size (p = 0.712), regional lymph nodes removed (p = 0.969), and surgery (p = 0.208) between the two groups.

**Table 1:** Association of tumor location with demographic and clinicopathological characteristics in patients.

N (%) variables	All patients	Prepuce/glans	Body of penis	p
No. of patients	455 (100.0)	409 (90.0)	46 (10.0)	
Age				0.278
<60	100 (22.0)	87 (21.3)	13 (28.3)	
≥60	355 (78.0)	322 (78.7)	33 (71.7)	
Ethnicity				**0.014**
Caucasian	371 (81.5)	337 (82.4)	34 (73.9)	
African-American	53 (11.6)	42 (10.3)	11 (23.9)	
Other	31 (6.8)	30 (7.3)	1 (2.2)	
T stage				0.853
Ta–T1	263 (57.8)	237 (57.9)	26 (56.5)	
T2–T4	192 (42.2)	172 (42.1)	20 (43.5)	
Lymph nodes status				**0.001**
N0	389 (85.5)	357 (87.3)	32 (69.6)	
N1–N3	66 (14.5)	52 (12.7)	14 (30.4)	
Grade				0.134
G1+G2	328 (72.1)	300 (73.3)	28 (60.9)	
G3+G4	74 (16.3)	62 (15.2)	12 (26.1)	
Unknown	53 (11.6)	47 (11.5)	6 (13.0)	
Tumor size				0.712
<3	204 (44.8)	186 (45.5)	18 (39.1)	
≥3	172 (37.8)	153 (37.4)	19 (41.3)	
Unknown	79 (17.4)	70 (17.1)	9 (19.6)	
Regional lymph nodes removed				0.969
No	365 (80.2)	328 (80.2)	37 (80.4)	
Yes	90 (19.8)	81 (19.8)	9 (19.6)	
Surgery				0.208
Non-radical surgery	141 (31.0)	123 (30.1)	18 (39.1)	
Radical surgery	314 (69.0)	286 (69.9)	28 (60.9)	

Significant values in bold.

**Figure 1:**
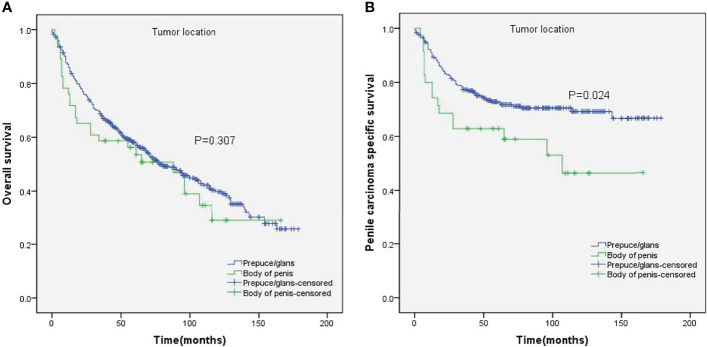
Kaplan–Meier analyses of overall survival **(A)** and penile carcinoma-specific survival **(B)** between different tumor locations.

The impact of tumor location on the OS and PCSS was evaluated by the Kaplan–Meier method (**Figure 1**). There was no significant difference between the OS of both groups (p = 0.307, **Figure 1A**). Nevertheless, PCSS was significantly higher for patients with prepuce/glans tumors compared to those with tumors in the body of the penis (p = 0.024, **Figure 1B**). As shown in **Table 2**, age (p < 0.001), T stage (p = 0.017), lymph nodes status (p < 0.001), grade (p = 0.003), tumor size (p = 0.003), and surgery (p = 0.025) were significantly associated with PCSS. The 5-year survival is also shown in **Table 2**. These variables were then incorporated into the Cox proportional hazards regression model for the multivariate analyses.

**Table 2:** Kaplan–Meier analyses predicting penile carcinoma-specific survival.

Variables	Penile carcinoma-specific survival, %	
	5-year rate (SEM)	p
Age		**<0.001**
<60	84.7 (4.1)	
≥60	66.4 (3.3)	
Ethnicity		0.673
Caucasian	71.7 (3.0)	
African-American	68.6 (7.8)	
Other	75.6 (9.5)	
T stage		**0.017**
Ta–T1	78.2 (3.4)	
T2–T4	63.8 (4.2)	
Lymph nodes status		**<0.001**
N0	76.3 (2.8)	
N1–N3	51.7 (6.8)	
Grade		**0.003**
G1+G2	71.5 (3.2)	
G3+G4	60.6 (7.0)	
Unknown	90.0 (5.5)	
Tumor size		**0.003**
<3	80.6 (3.5)	
≥3	60.5 (4.7)	
Unknown	71.7 (6.7)	
Regional lymph nodes removed		0.600
No	72.0 (3.1)	
Yes	70.4 (5.3)	
Surgery		**0.025**
Non-radical surgery	82.2 (4.3)	
Radical surgery	67.4 (3.3)	
Tumor location		**0.024**
Prepuce/glans	72.8 (2.8)	
Body of penis	62.9 (8.2)	

Significant values in bold.

**Figure 2:**
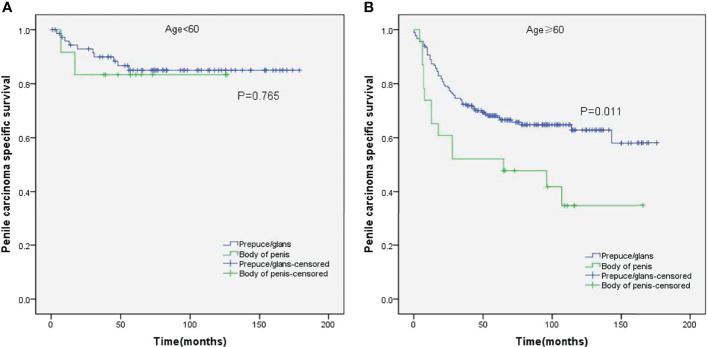
Kaplan–Meier analyses of penile carcinoma-specific survival in age < 60 years group **(A)** and age ≥ 60 years group **(B)** in patients stratified by tumor location.

**Figure 3:**
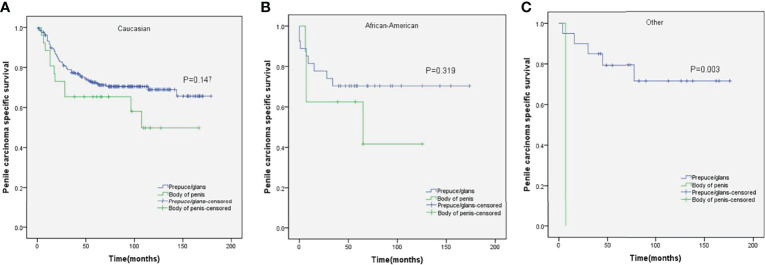
Kaplan–Meier analyses of penile carcinoma-specific survival in different ethnicities **(A–C)** in patients stratified by tumor location.

**Figure 4:**
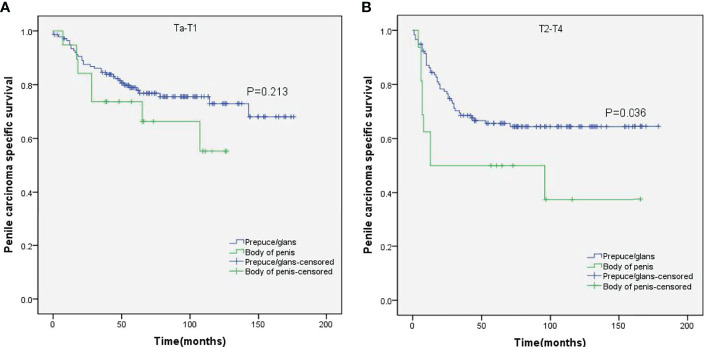
Kaplan–Meier analyses of penile carcinoma-specific survival on Ta–T1 group **(A)** and T2–T4 group **(B)** in patients stratified by tumor location.

**Figure 5:**
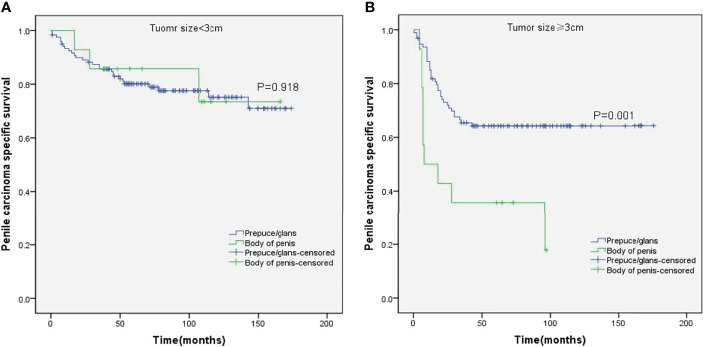
Kaplan–Meier analyses of penile carcinoma-specific survival on tumor size < 3 cm group **(A)** and tumor size ≥ 3 cm group **(B)** in patients stratified by tumor location.

**Figure 6:**
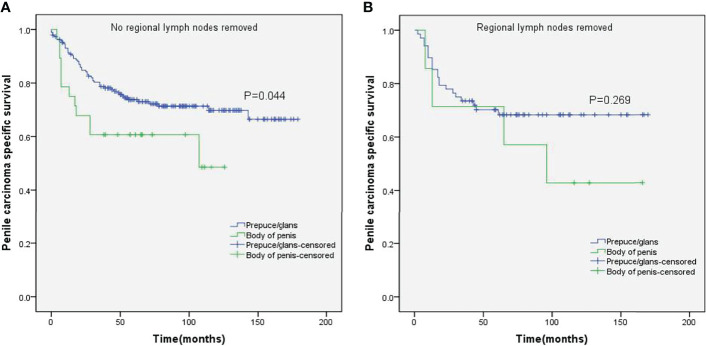
Kaplan–Meier analyses of penile carcinoma-specific survival on no regional lymph nodes removed group **(A)** and regional lymph nodes removed group **(B)** in patients stratified by tumor location.

**Figure 7:**
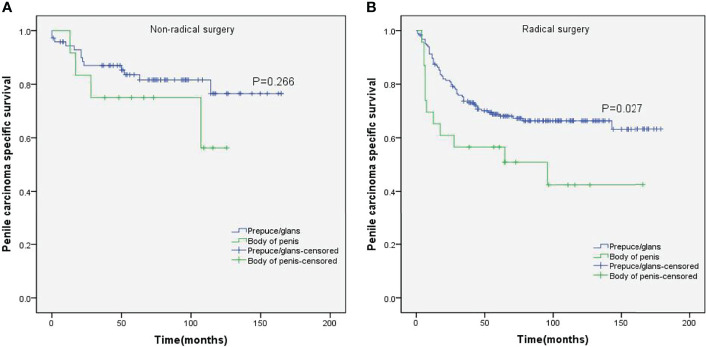
Kaplan–Meier analyses of penile carcinoma-specific survival in non-radical surgery group **(A)** and radical surgery group **(B)** in patients stratified by tumor location.

**Figure 8:**
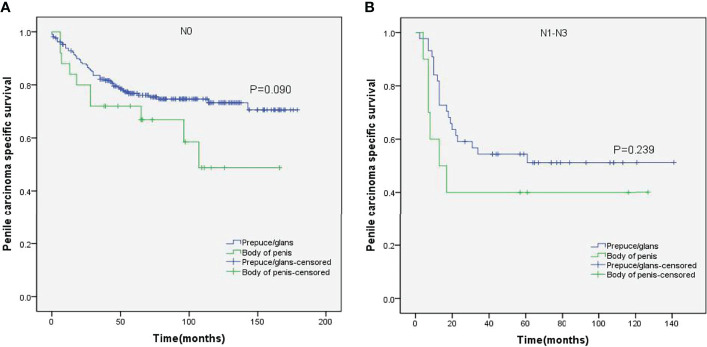
Kaplan–Meier analyses of penile carcinoma-specific survival in N0 group **(A)** and N1–N3 group **(B)** in patients stratified by tumor location.

**Figure 9:**
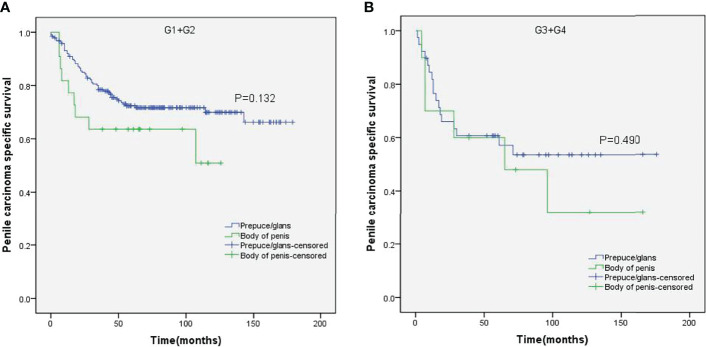
Kaplan–Meier analyses of penile carcinoma-specific survival in G1+G2 group **(A)** and G3+G4 group **(B)** in patients stratified by tumor location.

Stratified Kaplan–Meier analyses further revealed that compared to prepuce/glans tumors, M0 SCCP in the body of the penis was significantly associated with poorer PCSS in patients with age ≥ 60 years (p = 0.011, **Figure 2B**), other ethnicities (p = 0.003, **Figure 3C**), T2–T4 stage (p = 0.036, **Figure 4B**), tumor size ≥ 3 cm (p = 0.001, **Figure 5B**), no regional lymph nodes removed (p = 0.044, **Figure 6A**), and radical surgery (p = 0.027, **Figure 7B**). However, there were no statistical differences in PCSS of patients with age < 60 years (p = 0.765, **Figure 2A**), Caucasian (p = 0.147, **Figure 3A**), African-American (p = 0.319, **Figure 3B**), Ta –T1 stage (p = 0.213, **Figure 4A**), tumor size < 3 cm (p = 0.918, **Figure 5A**), regional lymph nodes removed (p = 0.269, **Figure 6B**), non-radical surgery (p = 0.266, Figure 7A), N0 stage (p = 0.090, **Figure 8A**), N1-N3 stage (p = 0.239, **Figure 8B**), G1+G2 (p = 0.132, **Figure 9A**), and G3+G4 (p = 0.490, **Figure 9B**) between the two groups.

As presented in **Table 3**, multivariate Cox proportional hazards analyses indicated that tumor location was an independent prognostic factor for PCSS (HR 1.881, p = 0.026). In addition, age (HR 3.336, p < 0.001), lymph node status (HR 2.530, p < 0.001), and tumor size (p = 0.045) were also independent predictors of PCSS. However, T stage (HR 1.097, p = 0.719), grade (p = 0.121), and surgery (HR 1.181, p = 0.598) were not significantly associated with PCSS.

**Table 3:** Multivariate Cox regression analyses predicting penile carcinoma-specific survival.

Variables		Penile carcinoma-specific survival	
		HR (95% CI)	p
Age		3.336 (1.798–6.190)	**<0.001**
T stage		1.097 (0.662–1.817)	0.719
Lymph nodes status		2.530 (1.557–4.109)	**<0.001**
Grade			0.121
G1+G2		Reference	**–**
G3+G4		1.423 (0.866–2.338)	0.164
Unknown		0.482 (0.169–1.375)	0.172
Tumor size			**0.045**
<3		Reference	–
≥3		1.854 (1.129–3.044)	**0.015**
Unknown		1.598 (0.862–2.964)	0.137
Surgery		1.181 (0.637–2.188)	0.598
Tumor location		1.881 (1.077–3.286)	**0.026**

Significant values in bold.

HR, hazard ratio.

## Discussion

Although SCCP is a relatively uncommon malignancy, its incidence has increased globally over the past decade (10). It is a painful disease associated with significantly high mortality rates (2). Due to uncontrollable local disease or distant metastasis, untreated SCCP patients usually die within 2 years of diagnosis (23). Therefore, it is necessary to explore the prognostic factors of SCCP in order to develop appropriate and individualized treatment plans. We conducted a retrospective study to determine the prognostic value of tumor location for M0 SCCP and found that tumors in the prepuce/glans had better PCSS than those in the penile body. Moreover, we identified tumor location as an independent prognostic factor for patients with M0 SCCP.

Previous studies have reported that SCCP is more prevalent in men between 50 and 70 years of age (6, 9). Consistent with this, SCCP was predominant in elderly men (age ≥ 60 years) in our study as well. Moreover, we also found that SCCP was more likely to occur in the prepuce or glans (90%). Masterson et al. also reported (24) that the most common site for penile cancer was the glans and/or prepuce, which accounted for 78% of the diagnosed cases. Furthermore, men of all ethnicities were susceptible to SCCP in the prepuce or glans.

While the location of M0 SCCP had no impact on the OS, the PCSS was significantly higher in patients with tumors in the prepuce or glans compared to those with tumors in the body of the penis. In addition, M0 SCCP in the body of the penis was more likely to metastasize to the lymph nodes. It is well known that lymph node metastasis is an important poor prognostic factor for SCCP (2, 25–27). Novara et al. also reported that the 5-year cancer-specific survival probabilities of clinically node-negative SCCP patients ranged from 75% to 93% and were significantly lower for those with node-positive patients (23). Moreover, the results of stratified Kaplan–Meier analyses indicated that tumors in the body of the penis were significantly associated with poorer PCSS in patients with no regional lymph node removed. However, no significant difference was observed between the PCSS of both groups in patients with regional lymph nodes removed. Based on these findings, we hypothesized that the higher incidence of lymph node metastasis in patients with M0 SCCP in the body of the penis was the major cause of poor PCSS. Furthermore, stratified Kaplan–Meier analyses showed that penile body tumors were associated with worse prognosis in patients with advanced T stage (T2–T4) and larger tumors (≥3 cm), which are significantly associated with lymph node metastasis in SCCP (20, 23). These findings further confirm our hypothesis.

Chaux et al. had proposed that the anatomical location of SCCP might have prognostic significance (28). But they did not conduct a comprehensive and detailed study. Excitingly, we have shown for the first time that tumor location is an independent prognostic factor for PCSS in patients with M0 SCCP, according to multivariate Cox proportional hazards analyses. Furthermore, we discovered that age was an independent predictor of PCSS. This discovery is also novel.

Although the surgery was not significantly associated with PCSS in multivariate Cox proportional hazards analyses, the Kaplan–Meier curves of the stratified analyses showed that prepuce/glans tumors were associated with better PCSS in patients who had undergone radical surgery. In contrast, tumor location had no significant impact on the PCSS of patients with non-radical surgery. Therefore, radical surgery can offer survival benefits for patients with M0 SCCP in the prepuce or glans.

Huang et al. reported that ethnicity was an independent predictor of PCSS (29). Conversely, the result of this study demonstrated that there was no significant difference among the PCSS of different ethnicities. In addition, stratified Kaplan–Meier analyses revealed that compared to prepuce/glans tumors, M0 SCCP in the body of the penis was significantly associated with poorer PCSS in patients of other ethnicities, but there was no statistical difference in the PCSS of different tumor locations in Caucasian patients of African-American patients. We speculated that this difference might be caused by unequal access to routine healthcare (30).

There are some limitations in our study that ought to be considered. First, the SEER data do not include family history and comorbidities, which limits the interpretation of factors related to survival. Second, data related to adjuvant chemotherapy and radiotherapy are also not provided by the SEER database. Third, all patients in this study were from the United States. Nevertheless, our findings can still be useful for the management of SCCP.

## Conclusions

Patients with M0 SCCP in the body of the penis were more likely to develop lymph node metastases than those with prepuce or glans tumors. Moreover, the PCSS was significantly better in patients with M0 SCCP in the prepuce or glans. The tumor location is an independent prognostic factor for M0 PCSS and should be considered during the clinical management.

## Data Availability Statement

Publicly available datasets were analyzed in this study. This data can be found here: https://seer.cancer.gov/.

## Ethics Statement

Approval of the Institutional Review Board and informed consent from patients were not required for the SEER database, as it is a de-identified public-use database.

## Author Contributions

KL, CF, and JS conceived and designed the project. KL, XL, and JW collected and analyzed the data. KL, XL, CF, and JS wrote the manuscript. All authors read and approved the final manuscript.

## Funding

This work was supported by a grant from Suzhou Municipal Health Commission (Grant number LCZX202010).

## Conflict of Interest

The authors declare that the research was conducted in the absence of any commercial or financial relationships that could be construed as a potential conflict of interest.

## Publisher’s Note

All claims expressed in this article are solely those of the authors and do not necessarily represent those of their affiliated organizations, or those of the publisher, the editors and the reviewers. Any product that may be evaluated in this article, or claim that may be made by its manufacturer, is not guaranteed or endorsed by the publisher.
